# Evaluating the prognostic value of mutational signatures in small-cell lung cancer through reference-based signature assignment and continuous activity analysis

**DOI:** 10.3389/fgene.2026.1776578

**Published:** 2026-07-20

**Authors:** Rishabh Garg, Kira A. Glasmacher, Arnaud Augert, Jeffrey P. Townsend

**Affiliations:** 1 Yale College, New Haven, CT, United States; 2 Department of Biostatistics, Yale School of Public Health, New Haven, CT, United States; 3 Program in Computational Biology and Biomedical Informatics, Yale University, New Haven, CT, United States; 4 Department of Pathology, Yale School of Medicine, Yale University, New Haven, CT, United States; 5 Yale Cancer Center, New Haven, CT, United States

**Keywords:** *de novo* extraction, immuno-oncology biomarkers, overall survival, reference-based signature assignment, single-base substitution 13 (SBS13), single-base substitution 4 (SBS4), small-cell lung cancer (SCLC), tumor mutational burden

## Abstract

**Background:**

Small-cell lung cancer (SCLC) is an aggressive malignancy with poor survival outcomes. Biomarkers that reliably capture tumor mutational burden (TMB), tumor immunogenicity, and prognosis could substantially improve clinical stratification. Prior studies have suggested that mutational signatures, including single-base substitution (SBS) 4 and 13, may be associated with TMB and survival in SCLC. We therefore evaluated these relationships using complementary analytical approaches.

**Methods:**

We evaluated associations among mutational signatures, TMB, and overall survival in a dataset examined in a prior study and an independent external cohort. To improve reproducibility and interpretability, we performed reference-based mutational signature assignment and analyzed signature activity, primarily as a continuous variable, while also evaluating binary stratifications using fixed thresholds. Signature–TMB relationships were assessed using continuous regression models, and associations with overall survival were evaluated pooling all clinically annotated samples using Kaplan-Meier analysis and Cox-proportional-hazards modeling.

**Results:**

Across both cohorts, SBS4 activity was positively associated with TMB, whereas SBS13 showed no consistent relationship with TMB. Neither SBS4 nor SBS13 was a statistically significant predictor of overall survival.

**Conclusion:**

Our study clarifies the prognostic and immunogenomic relevance of SBS4 and SBS13 in SCLC and shows that analytical choices materially influence inference. Reference-based signature assignment provided a comprehensive, computationally efficient, and stable framework, while continuous measures of signature activity yielded more reliable inferences than arbitrary thresholds. These findings support more rigorous evaluation of mutational signatures and TMB as biomarkers in SCLC.

## Introduction

Small-cell lung cancer (SCLC), comprising approximately 15% of all lung cancers, remains one of the most aggressive malignancies, with persistently poor prognosis (Rudin et al., 2021). Despite extensive investigation into candidate biomarkers, no molecular biomarker has yet been validated for routine clinical use in stratifying outcomes or guiding treatment decisions (Thomas et al., 2025). Recent work has proposed that mutational signatures, including single-base signature 4 (SBS4; smoking-associated) and SBS13 (APOBEC-mediated) may carry prognostic value in SCLC (Li et al., 2025), highlighting the potential of mutational processes as clinically informative biomarkers. Considering the growing interest in mutational signatures for translational oncology, it is important to assess the robustness and generalizability of these associations across analytical frameworks and independent cohorts. Here, we sought to evaluate the relationships between SBS4, SBS13, tumor mutational burden, and overall survival using complementary computational approaches, with the goal of clarifying their potential utility as prognostic biomarkers in SCLC.

Validated mutational biomarkers can guide treatment decisions, including tailoring therapy modality and intensity to individual patients. Indeed, TMB is increasingly recognized in both research and selected clinical contexts as a prognostic and predictive metric, with higher TMB often associated with improved responses to immune-checkpoint inhibitor (ICI) therapies in SCLC and other cancers (Marabelle et al., 2020; Hellmann et al., 2018). Recent work (Li et al., 2025) has suggested that high SBS4 may be associated with lower TMB and poorer survival. However, SBS4, the canonical signature of tobacco smoke mutagenesis (Alexandrov et al., 2016; Hecht, 2003)—a major etiologic factor in SCLC (Basumallik and Agarwal, 2025)—has been linked to higher TMB in lung cancers, including non-small cell lung cancer (Alexandrov et al., 2016; Tan et al., 2022) and SCLC (Wang et al., 2023).

Likewise, a reported association between high SBS13, elevated TMB, and better survival in the previous study (Li et al., 2025) merits further evaluation in light of treatment context. The cohort originally sequenced by George et al. (2015) and subsequently reanalyzed (Li et al., 2025) was composed predominantly of early-stage (I–III), treatment-naive tumors, with only a small number of cases sampled at relapse. In addition, George et al. (2015) did not report prior ICI treatment; and their study—published in 2015—preceded FDA approval of atezolizumab, nivolumab, and pembrolizumab for SCLC (George et al., 2015; Horn et al., 2018). In the absence of ICI therapy, high TMB has been associated with poorer survival in many non-ICI contexts (Valero et al., 2021)—likely due to high mutation rates resulting in better ability for the cancer to evolve under selective pressures and develop resistance to conventional therapies (Valero et al., 2021).

Together, these observations motivate further investigation into the mechanistic and associative relationships between mutational signatures, TMB, and overall survival in SCLC across analytical frameworks and datasets. We therefore conducted validatory and complementary analyses to assess the robustness and interpretability of associations between mutational signature activity, TMB, and clinical outcomes, with the goal of clarifying the prognostic utility of SBS4 and SBS13 in SCLC.

## Methods

### Data collection

We analyzed two SCLC whole-exome sequencing cohorts. The primary cohort was the dataset analyzed by Li et al. (2025), comprising 110 whole-exome-sequenced tumor samples, including 101 samples with accompanying clinical data from George et al. (2015) and 42 whole-exome sequenced tumor samples from Rudin et al. (2012). Because survival analyses required clinical annotation, the George et al. (2015) cohort served as the principal dataset for analyses relating mutational signatures to overall survival, whereas the combined George et al. (2015) + Rudin et al. (2012) dataset was used for analyses of mutational signature activity and tumor mutational burden, consistent with data availability.

For independent validation, we assembled a multi-study SCLC cohort comprising whole-exome sequencing from Jiang et al. (15; n = 98 tumors, including 95 with clinical annotation), Zhou et al. (16; n = 40 tumors with clinical annotation), Wang et al. (10; n = 177 tumors without clinical annotation), Chen et al. (17; n = 18 tumors with clinical annotation), and Liu et al. (18; n = 112 tumors, including 108 with clinical annotation). In total, this multi-study cohort contained 445 tumors, of which 362 featured accompanying clinical annotations for survival analyses. This cohort was used to evaluate the generalizability of associations between mutational signature activity, TMB, and overall survival. All data processing and statistical analyses were performed in R (v. 4.5.2) using processed mutation and clinical data extracted from the Supplementary Material of the original publications. Mutational signature attribution, threshold optimization, and survival analyses were then performed as described below.

### Signature assignment and exclusions

Reference-based mutational signature assignment was performed using 15 COSMIC (v3.2) signatures using MutationalPatterns in R, with primary analyses restricted to 15 signatures that are biologically plausible in SCLC and smoking-related lung tumorigenesis (SBS1–6, 13, 15, 16, 24, 29, 39, 40, 60, and 92), as guided by previous studies (George et al., 2024; Tang et al., 2022; Alexandrov et al., 2020). These signatures were selected to capture age-related, tobacco-associated, APOBEC-associated, mismatch repair-related, and other mutational processes plausibly contributing to SCLC genomes while limiting overfitting from inclusion of implausible signatures. For each dataset, signature attribution was performed separately for pre-treatment and post-treatment tumors. For post-treatment samples, treatment-associated signatures (SBS11, 31, 32, 35, 86, 87, and 90) were also included in the candidate signature set to account for mutations potentially induced by prior therapy and to reduce misattribution of treatment-related mutations to endogenous or tobacco-associated processes. These treatment-associated signatures were excluded from pre-treatment analyses, where prior therapy could not have contributed to mutagenesis. For cases lacking treatment information, treatment-associated signatures were retained to avoid excluding potentially relevant mutational processes. To ensure robustness of the deconvolution algorithm, all findings were independently re-evaluated using deconstructSigs (v1.8.0) (Rosenthal et al., 2016) as an alternative reference-based signature assignment method. Relatively flat, featureless signatures—such as SBS5 and SBS40—are challenging to attribute uniquely and were therefore interpreted with corresponding caution.

### Average proportional contribution of signatures to tumor mutational burden and total cancer effect

Tumor mutational burden (TMB) was defined as the total number of somatic single-nucleotide variants identified by whole-exome sequencing normalized to the exome coverage and expressed as mutations per megabase (mutations/Mb). For each tumor, mutational signature activity was calculated by reference-based attribution and expressed as the proportion of total single nucleotide variants assigned to each signature. To summarize cohort-level trends in mutagenesis, we calculated the average proportional contribution of each signature to TMB by averaging per-sample signature activities across all tumors.

We also quantified the average proportional contribution of each signature to total cancer effect—a quantitative measure of selective advantage conferred by a mutation to a cell—by averaging activities across all samples as in Cannataro et al. (2022). Total cancer effect represents the summed selective effects of somatic variants within a tumor, providing an estimate of the aggregate contribution of those variants to oncogenic cellular advantage. For each sample, we apportioned total cancer effect across signatures according to the inferred contribution of each signature to the variants present, and then averaged these proportions across the cohort. This procedure yielded a single normalized value per signature for its mean contribution to genome-wide mutagenesis and a corresponding normalized value for its mean contribution to total cancer effect in the cohort.

### Tumor mutational burden analysis

We performed three complementary analyses to evaluate the relationship between mutational signature activity and TMB. Because the distributions of TMB and signature activity were non-normal in both the Li et al. (2025) dataset (Shapiro-Wilk tests on TMB: *P* = 5.17 × 10^−7^; SBS4 signature activity: *P* = 8.44 × 10^−4^; and SBS13 signature activity: *P* < 2.2 × 10^−16^) and the independent validation cohort (Shapiro-Wilk tests on TMB: *P* = 3.59 × 10^−11^; SBS4 signature activity: *P* = 6.25 × 10^−10^; and SBS13 signature activity: *P* < 2.2 × 10^−16^), groupwise comparisons were assessed using Wilcoxon rank-sum tests. These analyses evaluated [1] the original 0.25 binary threshold applied to signature activities reported in Supplementary Table S3 of Li et al. (2025) [2] the same 0.25 threshold applied to reference-based derived signature activities inferred in the present study.

As a complementary continuous-scale analysis, we [3] fitted linear regression models relating continuous signature activity to log-transformed TMB and evaluated the regression slope using the two-sided *t*-test. Together, these analyses enabled us to compare the effects of previously used fixed thresholds, data-driven thresholds, and continuous representation of signature activity on inference regarding TMB associations.

### Survival analysis stratified by signature activity

Overall survival was defined as the interval from diagnosis to death from any cause, with patients alive at last follow-up treated as censored observations. We evaluated associations between mutational signature activity and overall survival using two pre-specified binary stratifications: [1] the original 0.25 activity threshold applied to the *de novo* signature activities reported in Supplementary Table S3 of Li et al. (2025), and [2] the same 0.25 threshold applied to reference-based signature activities inferred in the present study. These binary analyses were performed primarily to facilitate comparison with previously reported results.

For each stratification, survival functions were estimated using the Kaplan-Meier method and compared using two-sided log-rank tests. Effect sizes were quantified using Cox proportional-hazards models, reporting hazard ratios and 95% confidence intervals. Survival analyses were performed using the survival (v3.8-3) package and visualized with survminer (v0.5.0.999) in R. Because mutational signature activity is inherently continuous, our interpretation focused primarily on continuous activity analyses and their consistency across cohorts, with binary stratifications used as complementary analyses for comparison with prior work. As the study evaluated a small number of pre-specified, hypothesis-driven associations involving SBS4, SBS13, tumor mutational burden, and overall survival, no adjustment for multiple-testing was applied, and reported *P* values were interpreted directly.

## Results

**FIGURE 1:**
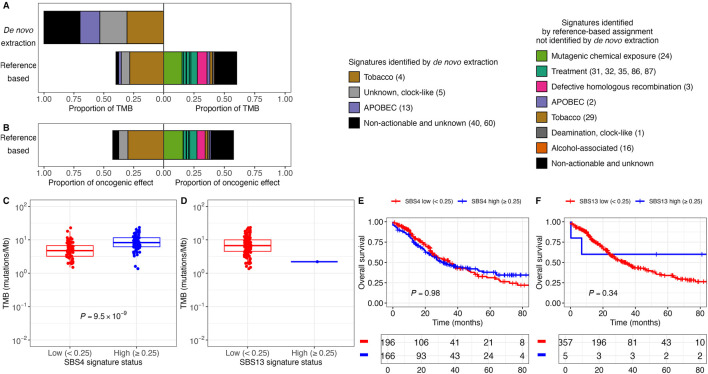
Differential tumor mutational burden (TMB) and overall survival between cohorts stratified by fixed thresholds of signature activities derived from reference-based assignment. **(A)** Average proportional contributions of mutational signatures to total TMB via *de novo* extraction by Li et al. (2025) (top) and via reference-based assignment (bottom). Five signatures identified by Li et al. (2025) (SBS4, 5, 13, 40, and 60) are depicted to the left of the center line with SBS40 and SBS60 included in the black label “non-actionable and unknown”; other signatures identified by reference-based assignment (SBS1, 2, 3, 6, 11, 15, 16, 24, 29, 31, 32, 35, 39, 86, 87, 90, and 92) are depicted to the right of the center line. **(B)** Proportions of oncogenic effect contributed by mutational signatures, identified and quantified by reference-based assignment. **(C)** Distribution of TMB between high and low SBS4 activity groups using a fixed threshold of 0.25 (*P* = 9.5 × 10^−9^; SBS4 high: *n* = 88; SBS4 low: *n* = 60; Hodges-Lehmann median difference: 130; 95% CI [90–170]). **(D)** Distribution of TMB between SBS13 subgroups using the same fixed threshold of 0.25 (SBS13 high: *n* = 1; SBS13 low: *n* = 147; insufficient samples over threshold for statistics). **(E)** Kaplan–Meier survival curves of overall survival stratified by SBS4 activity in the combined cohort of clinically annotated samples from the dataset analyzed by Li et al. (2025) and the external validation cohort (threshold = 0.25; *n* = 362; *P* = 0.98; HR = 1.0; 95% CI [0.75–1.3]). **(F)** Kaplan–Meier survival curves of overall survival stratified by SBS13 activity in the combined cohort of clinically annotated samples from the dataset analyzed by Li et al. (2025) and the external validation cohort (threshold = 0.25; *n* = 362; *P* = 0.34; HR = 0.51; 95% CI [0.13–2.1]). All *P* values reported for box plots were calculated using the Wilcoxon rank-sum test. All *P* values reported for Kaplan-Meier curves were calculated using the log-rank test.

Using two established reference-based methods (Rosenthal et al., 2016; Manders et al., 2022) on the 45,882 somatic variants from 152 SCLC whole exomes (Li et al., 2025) we identified numerous additional high-impact signatures contributing to SCLC mutagenesis beyond just the five COSMIC-match signatures identified through *de novo* signature extraction in Li et al. (2025) (SBS4: tobacco smoking; SBS5: clock-like, age-associated; SBS13: AID/APOBEC cytidine deaminases; SBS40: unknown etiology; and SBS60: known sequencing artefact; Figure 1A). Separation of background mutation rates from oncogenic selection (Mandell et al., 2023) revealed that these additional mutagenic processes not only generated mutations (Figure 1A), but also yielded substitutions with substantial oncogenic effects (Figure 1B).

To further assess associations between mutational signatures, TMB, and overall survival in SCLC, we re-analyzed the *de novo* signature activities and original data in Li et al. (2025), using their binary activity threshold of 0.25. With this threshold, tumors with high SBS4 activity exhibited significantly higher TMB than SBS4-low tumors (*P* = 8.1 × 10^−5^; Hodges-Lehmann median difference: 120; 95% CI: 66–180; Supplementary Figure S1A). In contrast, SBS13-high and -low tumors did not differ significantly in TMB (*P* = 0.88; Hodges-Lehmann median difference: −7; 95% CI: [−100−72]; Supplementary Figure S1B). Together, these results support a positive association between SBS4 activity and TMB in this cohort, while suggesting no clear association between SBS13 activity and TMB under the same thresholding framework.

We evaluated overall survival using the same Li et al. (2025) Kaplan-Meier stratification framework and the same 0.25 signature activity threshold applied by Li et al. (2025). Under this analysis, neither SBS4 nor SBS13 stratification yielded statistically significant differences in overall survival (Supplementary Figure S1C,D). Nevertheless, the survival curves showed consistent directional trends: the SBS4-high group exhibited lower survival probability at all time points compared to the SBS4-low group (Supplementary Figure S1C), and the SBS13-high group showed higher survival probability at all time points compared to the SBS13-low group (Supplementary Figure S1D). These findings suggest that although the direction of association was preserved, the corresponding survival differences were not statistically supported in our re-analysis under this thresholding framework.

To understand why equivalent analyses of ostensibly identical data yielded consistent directional trends but differing statistical significance, we examined data consistency across sources. We first confirmed that our dataset matched the original sequencing and clinical data from George et al. (2015). Then we compared these original annotations with those used in subsequent analyses (Li et al., 2025). This comparison identified discrepancies in survival status at last follow up between the original dataset (George et al., 2015) and the subsequent re-analysis (Li et al., 2025). These differences provide an unambiguous explanation for differences in statistical significance across analyses, highlighting the importance of consistent clinical annotation for survival-based inference.

Considering the methodological advantages of reference-based attribution for moderate-sized cohorts, we next re-analyzed the SCLC samples using MutationalPatterns, which provides a comprehensive, reproducible, and clinically feasible approach for mutational signature assignment. With this approach, high SBS4 activity (>0.25) was again associated with higher TMB (*P* = 9.5 × 10^–9^; Hodges-Lehmann median difference: 130; 95% CI [90–170]; Figure 1C), consistent with our analysis of the previously reported *de novo* signature activities. For SBS13, reference-based attribution yielded only a single sample surpassing the 0.25 threshold, precluding meaningful group comparison (Figure 1D). Together, these results support a robust positive association between SBS4 activity and TMB in this cohort, while indicating that the 0.25 threshold provides limited discriminatory value for SBS13 (Li et al., 2025).

We next examined whether either signature was associated with overall survival. To maximize statistical power, we pooled all clinically annotated tumors from the dataset analyzed by Li et al. (2025) (the George et al. (2015) cohort) with the clinically annotated samples of the external validation cohort, applying the 0.25 activity threshold across the combined dataset (*n* = 362). In this pooled cohort, high SBS4 activity was not associated with overall survival (*P* = 0.98; HR = 1.0; 95% CI [0.75–1.3]; Figure 1E), and high SBS13 activity likewise showed no association with overall survival (*P* = 0.34; HR = 0.51; 95% CI [0.13–2.1]; Figure 1F). Cohort-level analyses were concordant: stratification by SBS4 activity yielded no consistent survival difference across the five clinically annotated cohorts, with only one of five reaching statistical significance (Supplementary Figure S2), and SBS13-high tumors were too rare for reliable estimation in most cohorts and absent entirely in two (Supplementary Figure S3). We found that neither signature showed a clear or reproducible relationship with overall survival in SCLC.

**FIGURE 2:**
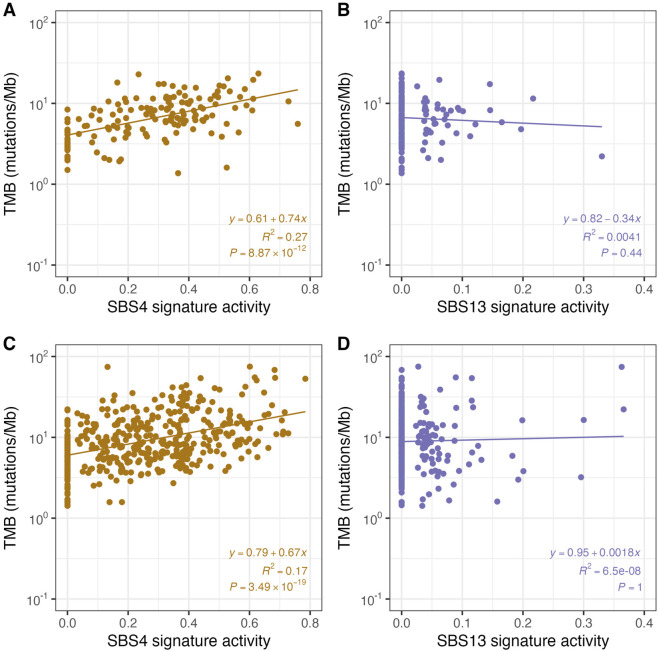
Continuous relationships between mutational signature activity and tumor mutational burden across cohorts. **(A)** Linear relationship between SBS4 signature activity and log_10_-transformed TMB using reference-based assignment of the whole-exome data analyzed by Li et al. (2025) (slope: 0.74; *t*-test *P* = 8.87 × 10^−12^; *R* ^2^ = 0.27). **(B)** Relationship between SBS13 activity and log_10_-transformed TMB of the whole-exome data analyzed by Li et al. (2025) (slope: −0.34; *t*-test *P* = 0.44; *R* ^2^ = 0.0041). **(C)** Relationship between SBS4 activity and log_10_-transformed TMB of the external validation cohort (slope: 0.67; *t*-test *P* = 3.49 × 10^−19^; *R* ^2^ = 0.17). **(D)** Relationship between SBS13 activity and log_10_-transformed TMB of the external validation cohort (slope: 0.0018; *t*-test *P* = 1; *R* ^2^ = 6.5 × 10^−8^). All slopes were evaluated using a two-sided *t*-test.

We next evaluated the relationship between mutational signature activities and TMB on a continuous scale based on reference-based signature assignments. SBS4 activity exhibited a strong positive linear relationship with TMB (slope: 0.74; *P* = 8.9 × 10^−12^; Figure 2A), consistent with the results obtained from binary stratification analyses and reinforcing the conclusion that higher SBS4 activity is associated with higher TMB. In contrast, SBS13 activity showed no significant linear association with TMB (slope: −0.34; *P* = 0.44; Figure 2B). These findings indicate that the association between SBS4 activity and TMB is robust across analytical frameworks, providing consistent evidence that there is no clear linkage between high SBS13 and elevated TMB.

To evaluate the generalizability of these findings, we applied the same reference-based continuous-activity analyses to an independent external SCLC cohort comprising tumor sequence data from (Wang et al., 2023; Jiang et al., 2016; Zhou et al., 2021; Chen et al., 2021; Liu et al., 2024). In this larger cohort, SBS4 activity exhibited a strong positive association with TMB (slope = 0.67; *R* ^2^ = 0.17; *P* = 3.49 × 10^−19^; Figure 2C), reproducing and strengthening the relationship observed in the dataset analyzed by Li et al. (2025). In contrast, SBS13 activity showed no detectable relationship with TMB (slope = 0.0018; *R* ^2^ = 6.5 × 10^−8^; *P* = 1; Figure 2D). Thus, analyses of an independent cohort supported the reproducibility of the SBS4–TMB association and provided no evidence for a meaningful association between SBS13 and TMB.

## Discussion

Our analysis of SCLC whole-exome and clinical data, integrating previously reported *de novo* mutational signature activities (Li et al., 2025) with reference-based mutational signature assignment, provides a re-evaluation of relationships among mutational signatures, TMB, and overall survival. Across datasets and analytical approaches, higher SBS4 activity was consistently associated with higher TMB, a finding opposite to the negative association reported previously (Li et al., 2025). This relationship is biologically plausible, considering the tobacco-related etiology of SBS4 and its previous linkages to elevated TMB in lung cancers (Valero et al., 2021). In contrast, SBS13 activity showed no consistent relationship with TMB across cohorts or analytical frameworks.

We found no clear or reproducible association between either SBS4 or SBS13 activity and overall survival (cf. Li et al., 2025). To maximize statistical power, survival analyses were performed using all clinically annotated tumors available across cohorts, yet neither signature was significantly associated with survival in the combined analysis. Consistent findings across cohort-specific and pooled analyses suggest that any survival effects of SBS4 or SBS13 are likely to be modest relative to currently available sample sizes. Substantially larger SCLC cohorts, particularly those with detailed treatment annotation and greater representation of high-SBS13 tumors, will be required to determine whether mutational signatures provide independent prognostic information beyond their association with mutational burden.

Our analyses support two methodological practices with broad relevance for mutational signature research. First, although *de novo* signature extraction can reveal previously unrecognized mutational patterns, it is better suited to larger cohorts, where greater statistical power reduces misassignment, particularly between closely related or relatively flat trinucleotide signatures (Pancotti et al., 2023). In the present study, reference-based signature assignment provided a more reproducible results than *de novo* extraction on small datasets, while also being more computationally accessible and enabling stable signature definitions (Wu et al., 2023) —crucial features for clinical translation. Second, representing signature activity as a continuous variable, rather than dichotomizing it at a fixed threshold, avoids arbitrary discretization and improves statistical power. Indeed, applying a binary threshold to a naturally continuous parameter is often arbitrary and problematic (Cohen, 1983; Giannoni et al., 2014). These practices enhance both the robustness of biomarker discovery and the potential clinical utility of mutational signatures by enabling more reliable prognostic stratification.

Building on these insights, we advocate for more powerful workflows that maximize rigor and translational potential. For TMB analyses: [1] assign mutational signatures using reference-based methods; [2] evaluate associations using continuous measures of signature activity, with fixed thresholds reserved for descriptive comparison; and [3] validate findings in independent cohorts. For survival analysis: [1] assign signatures using reference-based methods; [2] stratify patients by treatment type and status before analysis; [3] test survival associations within treatment-homogeneous groups using continuous measures of signature activity where possible, and [4] confirm results in external cohorts with comparable treatment data. Adoption of these practices will enhance the reproducibility, interpretability, and clinical applicability of mutational signature biomarkers in SCLC and more broadly in translational oncology.

## Data Availability

All analyses and figures are fully reproducible using the code and data processing scripts available at https://github.com/rishug123/SCLC-Commentary.

## Supplementary material

Supplementary figure S1 Binary signature activity analyses, using mutational activities reported by Li et al.^3^ and a previously proposed threshold of 0.25, dividing signature activity into high (blue) and low (red). **(A)** Tumor Mutational Burden (TMB) in the low-SBS4 (red, *n* = 33) and high-SBS4 (blue, *n* = 67) strata of the whole-exome dataset (Wilcoxon rank-sum test of difference *P* = 8.1 × 10^−5^; Hodges-Lehmann median difference: 120; 95% CI [66–180]; cf. Fig. 3E in Li et al. ^3^). **(B)** TMB in the low-SBS13 (red, *n* = 5) and high-SBS13 (blue, *n* = 15) strata of the whole-exome dataset (Wilcoxon rank-sum test of difference *P* = 0.88; Hodges-Lehmann median difference: −7; 95% CI [−100–72]; *cf.* Fig. 3F in Li et al. ^3^). **(C)** Kaplan–Meier survival curves of overall survival in patients from the Li et al. ^3^ whole-exome cohort stratified by SBS4 activity (log-rank test of difference *P* = 0.11; HR = 1.55, 95% CI [0.90–2.66]; *cf.* Fig. 2D in Li et al. ^3^). **(D)** Kaplan–Meier survival curves of overall survival in patients from the Li et al. ^3^ whole-exome cohort, stratified by SBS13 activity (log-rank test of difference *P* = 0.11; HR = 0.56, 95% CI [0.27–1.17]; *cf.* Fig 2F in Li et al. ^3^).

Supplementary figure s2 Kaplan–Meier survival curves of overall survival stratified by SBS4activity (threshold = 0.25; SBS4‐high: blue and SBS4‐low: red) within each clinically annotated cohort comprising the combined dataset. **(A)** Chen et al. (HR = 0.61; 95% CI [0.14–2.5]; log‐rank *P* = 0.49; *n* = 18). **(B)** George et al. (HR = 1.6; 95% CI [0.95–2.6]; log‐rank test ofdifference *P* = 0.079; *n* = 101). **(C)** Jiang et al. (HR = 1.1; 95% CI [0.55–2.4]; log‐rank *P* = 0.71; *n* = 95). **(D)** Liu et al. (HR = 0.57; 95% CI [0.34–0.97]; log‐rank *P* = 0.034; *n* = 108). **(E)** Zhou et al. (HR = 0.97; 95% CI [0.35–2.7]; log‐rank *P* = 0.96; *n* = 40).

Supplementary figure s3 Kaplan–Meier survival curves of overall survival stratified by SBS13 activity (threshold = 0.25; SBS13‐high: blue and SBS13‐low: red) within each clinically annotated cohort comprising the combined dataset. The clinically annotated cohorts of Chen et al. and Jiang et al. contained no SBS13‐high tumors (no sample with SBS13 activity > 0.25)and are therefore omitted. **(A)** George et al. (*n* = 101; log‐rank test of difference *P* = 0.12; the two SBS13‐high tumors were subject to no events, precluding a stable hazard‐ratio estimate). **(B)** Liu et al. (HR = 1.0; 95% CI [0.14–7.3]; log‐rank *P* = 1; *n* = 108). **(C)** Zhou et al. (HR = 1.2; 95% CI [0.1–9.5]; log-rank *P* = 0.86; *n* = 40).

## References

[B1] Alexandrov L. B. Ju Y. S. Haase K. Van Loo P. Martincorena I. Nik-Zainal S. (2016). Mutational signatures associated with tobacco smoking in human cancer. Science 354 (6312), 618–622. 10.1126/science.aag0299 27811275 PMC6141049

[B2] Alexandrov L. B. Kim J. Haradhvala N. J. Huang M. N. Tian Ng A. W. Wu Y. (2020). The repertoire of mutational signatures in human cancer. Nature 578 (7793), 94–101. 10.1038/s41586-020-1943-3 32025018 PMC7054213

[B3] Basumallik N. Agarwal M. (2025). “Small cell lung cancer,” in StatPearls (Treasure Island (FL): StatPearls Publishing).

[B4] Cannataro V. L. Mandell J. D. Townsend J. P. (2022). Attribution of cancer origins to endogenous, exogenous, and preventable mutational processes. Mol. Biol. Evol. 39 (5), msac084. 10.1093/molbev/msac084 35580068 PMC9113445

[B5] Chen M. Chen R. Jin Y. Li J. Hu X. Zhang J. (2021). Cold and heterogeneous T cell repertoire is associated with copy number aberrations and loss of immune genes in small-cell lung cancer. Nat. Commun. 12 (1), 6655. 10.1038/s41467-021-26821-8 34789716 PMC8599854

[B6] Cohen J. (1983). The cost of dichotomization. Appl. Psychol. Meas. 7 (3), 249–253. 10.1177/014662168300700301

[B7] George J. Lim J. S. Jang S. J. Cun Y. Ozretić L. Kong G. (2015). Comprehensive genomic profiles of small cell lung cancer. Nature 524 (7563), 47–53. 10.1038/nature14664 26168399 PMC4861069

[B8] George J. Maas L. Abedpour N. Cartolano M. Kaiser L. Fischer R. N. (2024). Evolutionary trajectories of small cell lung cancer under therapy. Nature 627 (8005), 880–889. 10.1038/s41586-024-07177-7 38480884 PMC10972747

[B9] Giannoni A. Baruah R. Leong T. Rehman M. B. Pastormerlo L. E. Harrell F. E. (2014). Do optimal prognostic thresholds in continuous physiological variables really exist? Analysis of origin of apparent thresholds, with systematic review for peak oxygen consumption, ejection fraction and BNP. PLoS One 9 (1), e81699. 10.1371/journal.pone.0081699 24475020 PMC3903471

[B10] Hecht S. S. (2003). Tobacco carcinogens, their biomarkers and tobacco-induced cancer. Nat. Rev. Cancer 3 (10), 733–744. 10.1038/nrc1190 14570033

[B11] Hellmann M. D. Callahan M. K. Awad M. M. Calvo E. Ascierto P. A. Atmaca A. (2018). Tumor mutational burden and efficacy of nivolumab monotherapy and in combination with ipilimumab in small-cell lung cancer. Cancer Cell 33 (5), 853–861. 10.1016/j.ccell.2018.04.001 29731394 PMC6750707

[B12] Horn L. Mansfield A. S. Szczęsna A. Havel L. Krzakowski M. Hochmair M. J. (2018). First-line atezolizumab plus chemotherapy in extensive-stage small-cell lung cancer. N. Engl. J. Med. 379 (23), 2220–2229. 10.1056/nejmoa1809064 30280641

[B13] Jiang L. Huang J. Higgs B. W. Hu Z. Xiao Z. Yao X. (2016). Genomic landscape survey identifies SRSF1 as a key oncodriver in small cell lung cancer. PLoS Genet. 12 (4), e1005895. 10.1371/journal.pgen.1005895 27093186 PMC4836692

[B14] Li Y. Song C. Wang H. Di W. Chen Y. Hu Y. (2025). Novel prognostic biomarkers in small cell lung cancer reveal mutational signatures, genomic mutations, and immune implications. Sci. Rep. 15 (1), 15592. 10.1038/s41598-025-00222-z 40320401 PMC12050310

[B15] Liu Q. Zhang J. Guo C. Wang M. Wang C. Yan Y. (2024). Proteogenomic characterization of small cell lung cancer identifies biological insights and subtype-specific therapeutic strategies. Cell 187 (1), 184–203.e28. 10.1016/j.cell.2023.12.004 38181741

[B16] Mandell J. D. Cannataro V. L. Townsend J. P. (2023). Estimation of neutral mutation rates and quantification of somatic variant selection using cancereffectsizeR. Cancer Res. 83 (4), 500–505. 10.1158/0008-5472.can-22-1508 36469362 PMC9929515

[B17] Manders F. Brandsma A. M. de Kanter J. Verheul M. Oka R. van Roosmalen M. J. (2022). MutationalPatterns: the one stop shop for the analysis of mutational processes. BMC Genomics 23 (1), 1–18. 10.1186/s12864-022-08357-3 35168570 PMC8845394

[B18] Marabelle A. Fakih M. Lopez J. Shah M. Shapira-Frommer R. Nakagawa K. (2020). Association of tumour mutational burden with outcomes in patients with advanced solid tumours treated with pembrolizumab: prospective biomarker analysis of the multicohort, open-label, phase 2 KEYNOTE-158 study. Lancet Oncol. 21 (10), 1353–1365. 10.1016/S1470-2045(20)30445-9 32919526

[B19] Pancotti C. Rollo C. Birolo G. Benevenuta S. Fariselli P. Sanavia T. (2023). Unravelling the instability of mutational signatures extraction *via* archetypal analysis. Front. Genet. 13, 1049501. 10.3389/fgene.2022.1049501 36685831 PMC9846778

[B20] Rosenthal R. McGranahan N. Herrero J. Taylor B. S. Swanton C. (2016). deconstructSigs: delineating mutational processes in single tumors distinguishes DNA repair deficiencies and patterns of carcinoma evolution. Genome Biol. 17 (1), 1–11. 10.1186/s13059-016-0893-4 26899170 PMC4762164

[B21] Rudin C. M. Durinck S. Stawiski E. W. Poirier J. T. Modrusan Z. Shames D. S. (2012). Comprehensive genomic analysis identifies SOX2 as a frequently amplified gene in small-cell lung cancer. Nat. Genet. 44 (10), 1111–1116. 10.1038/ng.2405 22941189 PMC3557461

[B22] Rudin C. M. Brambilla E. Faivre-Finn C. Sage J. (2021). Small-cell lung cancer. Nat. Reviews Dis. Primers 7 (1), 3. 10.1038/s41572-020-00235-0 33446664 PMC8177722

[B23] Tan C. Mandell J. D. Dasari K. Cannataro V. L. Alfaro-Murillo J. A. Townsend J. P. (2022). Heavy mutagenesis by tobacco leads to lung adenocarcinoma tumors with KRAS G12 mutations other than G12D, leading KRAS G12D tumors-on average-to exhibit a lower mutation burden. Lung Cancer 166, 265–269. 10.1016/j.lungcan.2021.10.008 34736794

[B24] Tang N. Li Z. Han X. Zhao C. Guo J. Wang H. (2022). Whole-exome sequencing uncovers specific genetic variation difference based on different modes of drug resistance in small cell lung cancer. Front. Oncol. 12, 891938. 10.3389/fonc.2022.891938 35847960 PMC9280676

[B25] Thomas A. Mohindroo C. Giaccone G. (2025). Advancing therapeutics in small-cell lung cancer. Nat. Cancer 6 (6), 938–953. 10.1038/s43018-025-00996-1 40523990

[B26] Valero C. Lee M. Hoen D. Wang J. Nadeem Z. Patel N. (2021). The association between tumor mutational burden and prognosis is dependent on treatment context. Nat. Genet. 53 (1), 11–15. 10.1038/s41588-020-00752-4 33398197 PMC7796993

[B27] Wang H. Wu S. Li Z. Zhang C. Shang X. Zhao C. (2023). Molecular subtyping of small-cell lung cancer based on mutational signatures with different genomic features and therapeutic strategies. Cancer Sci. 114 (2), 665–679. 10.1111/cas.15606 36178064 PMC9899606

[B28] Wu A. J. Perera A. Kularatnarajah L. Korsakova A. Pitt J. J. (2023). Mutational signature assignment heterogeneity is widespread and can be addressed by ensemble approaches. Brief. Bioinform 24 (6), bbad331. 10.1093/bib/bbad331 37742051 PMC10518036

[B29] Zhou H. Hu Y. Luo R. Zhao Y. Pan H. Ji L. (2021). Multi-region exome sequencing reveals the intratumoral heterogeneity of surgically resected small cell lung cancer. Nat. Commun. 12 (1), 5431. 10.1038/s41467-021-25787-x 34521849 PMC8440529

