# Supplementary figures and images for: Evaluating the prognostic value of mutational signatures in small-cell lung cancer through reference-based signature assignment and continuous activity analysis
Source: Front Genet. 2026 Jul 20;17:1776578. doi: 10.3389/fgene.2026.1776578 (PMC13429233; doi:10.3389/fgene.2026.1776578)

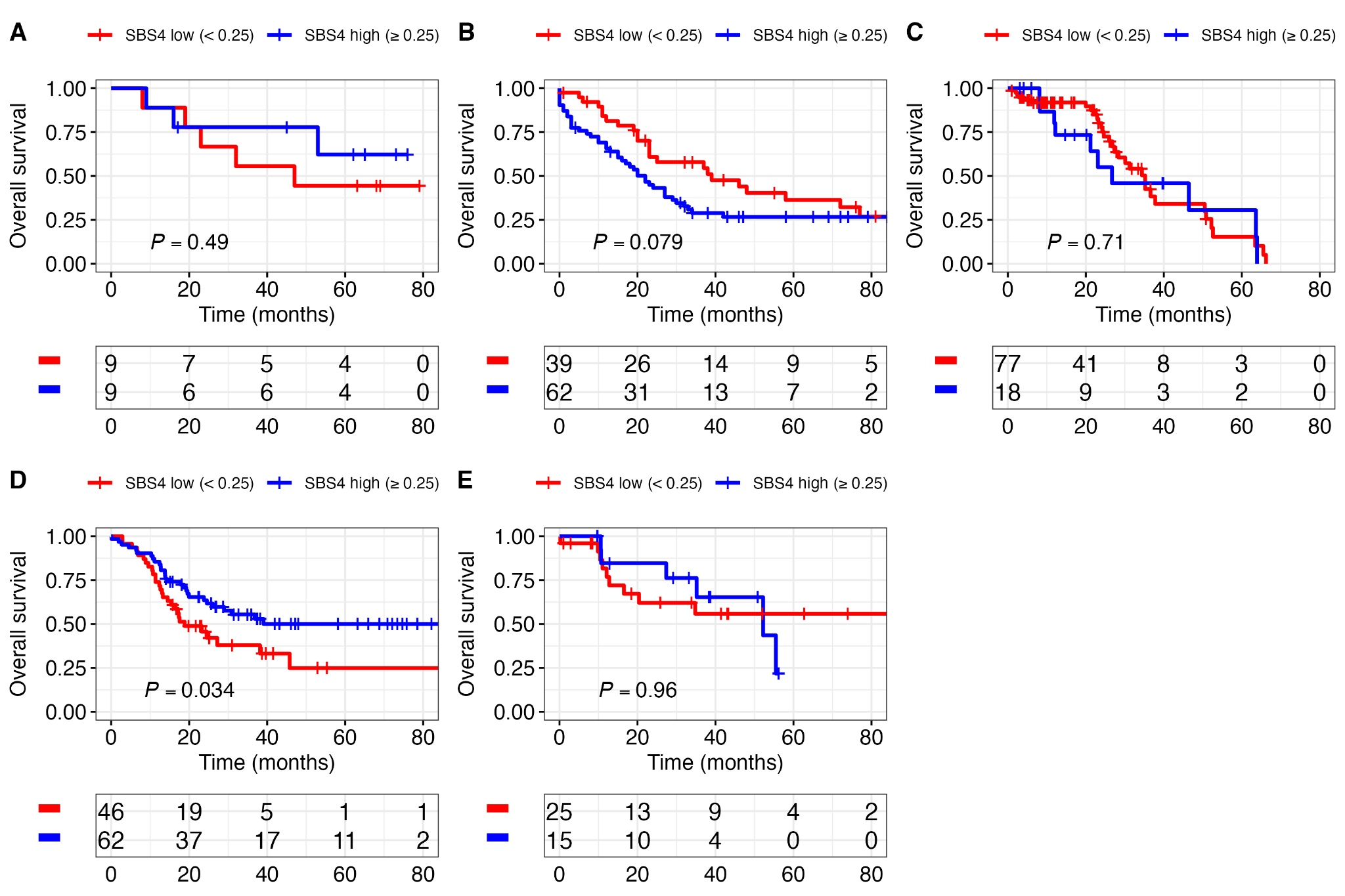

Supplement: Supplementary file 1 [file Image2.png]

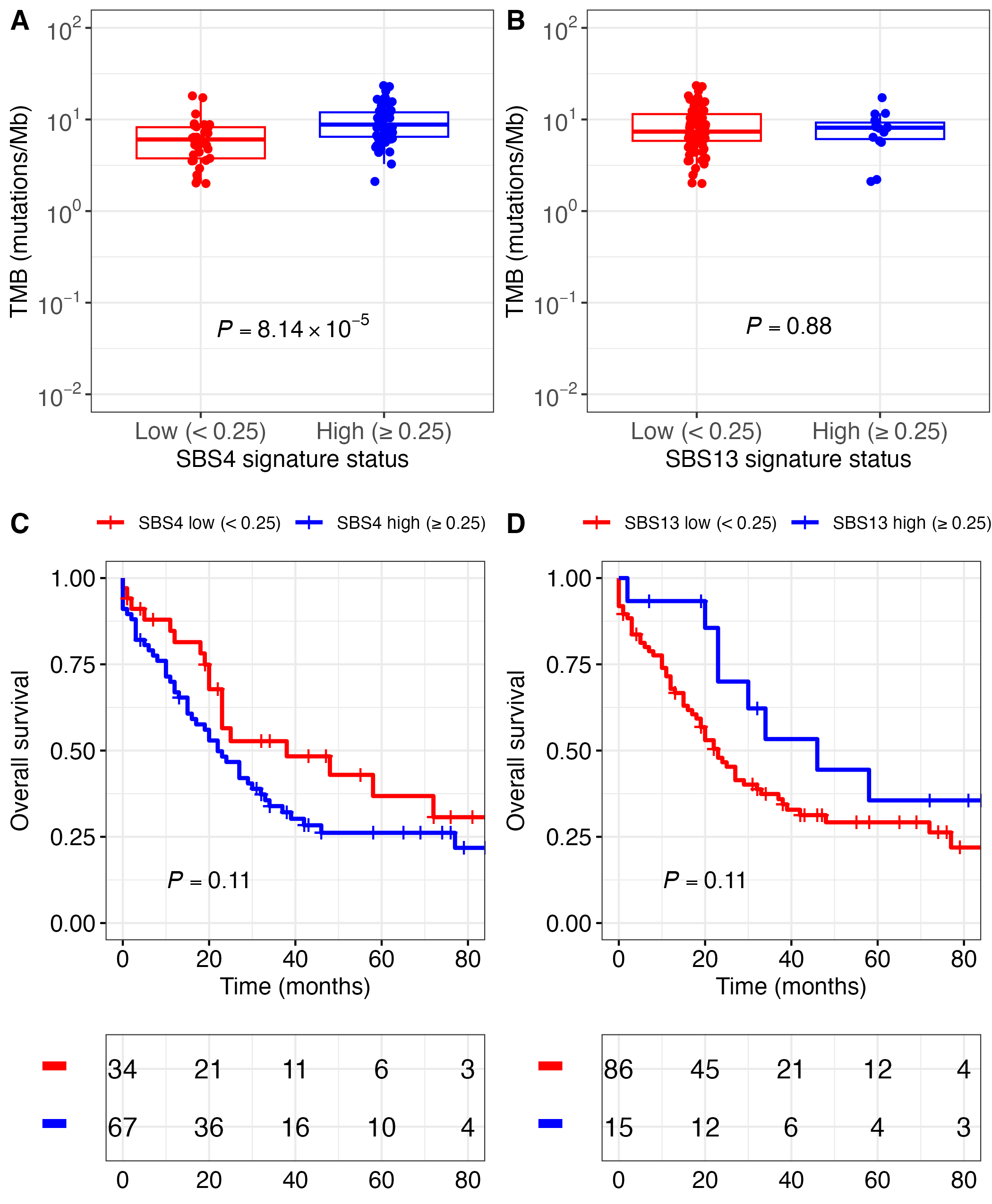

Supplement: Supplementary file 2 [file Image1.png]

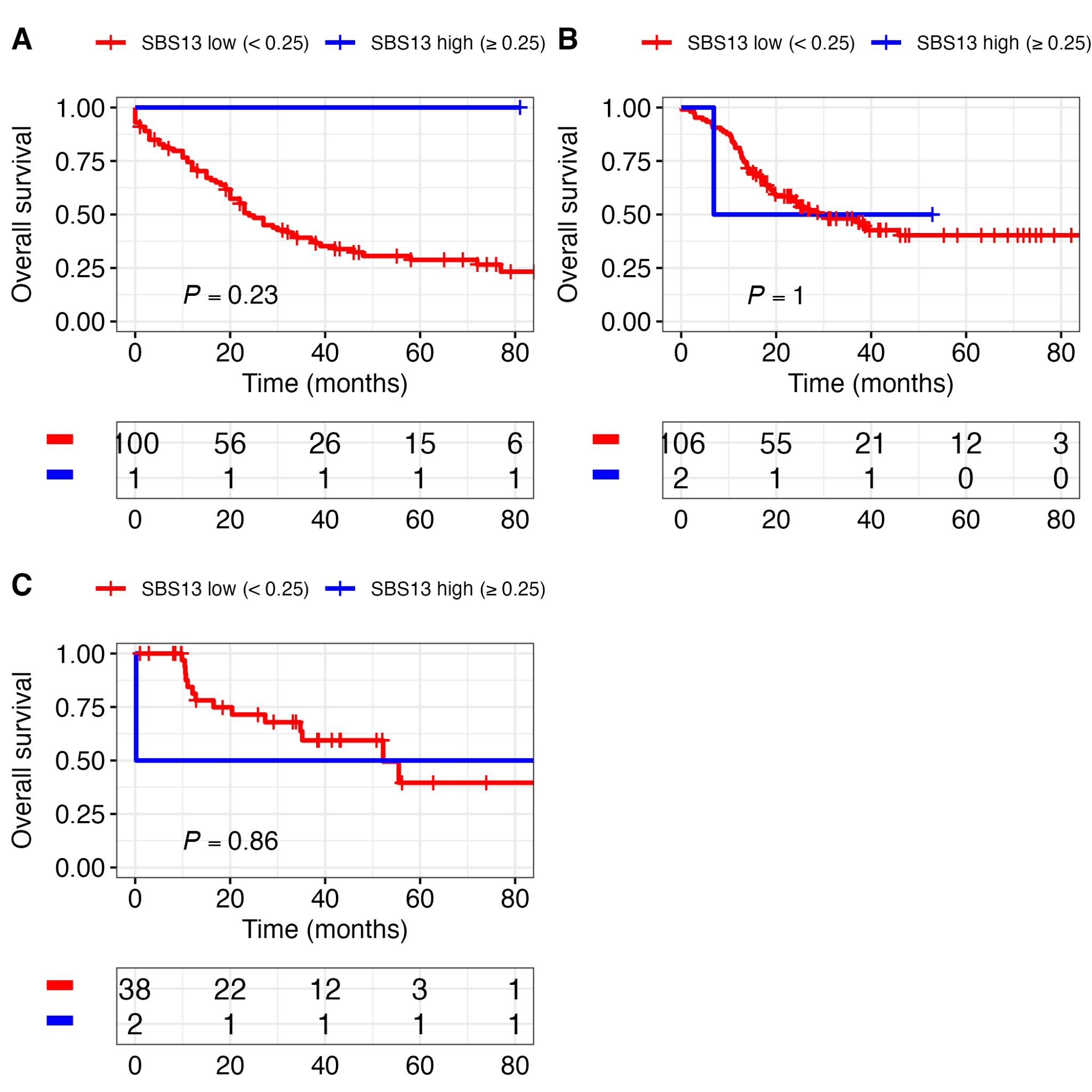

Supplement: Supplementary file 3 [file Image3.png]
